# Coinfection of *Gynura bicolor* with a New Strain of Vanilla Distortion Mosaic Virus and a Novel Maculavirus in China

**DOI:** 10.3390/v17101290

**Published:** 2025-09-24

**Authors:** Zhengnan Li, Mengze Guo, Pingping Sun, Lei Zhang

**Affiliations:** College of Horticulture and Plant Protection, Inner Mongolia Agricultural University, Hohhot 010018, China; modeiguo@gmail.com (M.G.); sunpingping@imau.edu.cn (P.S.); lei.zhang@imau.edu.cn (L.Z.)

**Keywords:** *Gynura bicolor*, high-throughput sequencing, vanilla distortion mosaic virus, maculavirus

## Abstract

In recent years, symptoms suggestive of viral infection have commonly occurred in *Gynura bicolor* in China. However, no viral genome infecting *G. bicolor* has been reported. This study applied high-throughput sequencing to plant samples with chlorotic spots in Sanya, Hainan. Viral sequences were confirmed using RT-PCR and RACE. Complete genomes of vanilla distortion mosaic virus (VDMV, *Potyvirus vanillae*) and an unknown virus were obtained. Sequence analysis indicated that the VDMV isolate from the *G. bicolor* is a novel variant. It shares 81.13% identity with its closest known strain. The unknown virus is phylogenetically related to maculaviruses but shares less than 76% nucleotide identity with other tymovirids. According to the ICTV, it should be classified as a new member of the genus *Maculavirus*. In this study, we provisionally designated the virus as gynura bicolor maculavirus (GBMV). Transmission electron microscopy revealed both filamentous and icosahedral virions in stems, but only filamentous virions in leaves. Quantitative RT-PCR showed high RNA accumulation of both viruses in the stems. GBMV levels were significantly lower in leaves. Dodder-mediated mechanical transmission successfully transferred VDMV and GBMV to *Nicotiana occidentalis*, *Oenothera biennis*, and *Chenopodium amaranticolor*. *O. biennis* developed chlorotic symptoms 15 days after dual infection.

## 1. Introduction

*Gynura bicolor*, a perennial herbaceous plant, is extensively cultivated across East and Southeast Asia for its nutritional and medicinal benefits [1,2]. The leaves of this plant are rich in anthocyanins, flavonoids, and phenolic compounds, which underpin its pronounced antioxidant and anti-inflammatory properties [3,4]. Recently, its cultivation has expanded in China. Concurrently, viral disease-like symptoms have become more common. No viral pathogens that infect *G. bicolor* have been identified [2,3]. Therefore, pathogen detection using high-throughput sequencing (HTS) is crucial for epidemiological surveillance and disease control.

The genus *Potyvirus* (family *Potyviridae*) is the largest group of plant viruses, comprising 183 recognized species [5]. Potyviruses infect diverse crops, including cereals and horticultural species, causing substantial economic losses. Infected plants frequently display mosaic, mottling, or necrotic symptoms [6,7,8,9,10]. Potyviruses possess a positive-sense, single-stranded RNA genome that encodes a polyprotein. This polyprotein is processed into multiple functional proteins by proteolytic cleavage [11]. The potyviral virions are flexuous filaments, 650–950 nm long and 11–20 nm in diameter. Potyviruses often co-infect plants with other viruses, producing synergistic effects that intensify disease severity. For instance, co-infection of maize by sugarcane mosaic virus (SCMV, *Potyvirus sacchari*) and maize chlorotic mottle virus (MCMV, *Machlomovirus zeae*) causes maize lethal necrosis disease, which is far more damaging than either infection alone [12,13].

The members of the genus *Maculavirus* (family *Tymoviridae*) are positive-sense, single-stranded RNA viruses that are strictly confined to the phloem of infected hosts. The viruses cannot be transmitted by sap inoculation. Virions are icosahedral with T = 3 symmetry, measuring about 30 nm in diameter. The viral genomes exhibit a notably high cytosine content, ranging from 32% to 50% [14,15,16]. The viral genome usually contains four ORFs. ORF1 encodes a polyprotein with conserved domains of methyltransferase, protease, helicase, and RNA-dependent RNA polymerase. ORF2 encodes the capsid protein, while ORF3 and ORF4 encode hypothetical proteins of unknown function [17,18,19]. Among maculaviruses, grapevine fleck virus (GFkV, *Maculavirus vitis*) and grapevine red globe virus (GRGV) cause leaf spots, vein clearing, and chlorosis, severely reducing grape quality and yield [19,20,21]. To date, no biological vector of maculaviruses has been identified. However, the viruses can be transmitted through infected propagative materials.

## 2. Materials and Methods

### 2.1. Plant Material

In December 2023, symptomatic leaves and stems of ten *G. bicolor* samples exhibiting chlorosis and mottling were collected from Sanya City, Hainan Province, China (18.2547° N, 109.5076° E). The plant leaves were flash-frozen in liquid nitrogen and stored at −80 °C for RNA extraction. The plant stems were preserved through vegetative propagation using stem cuttings.

### 2.2. High-Throughput Sequencing and De Novo Transcriptome Assembly

According to the manufacturer’s instructions, total RNA was extracted from 10 *G. bicolor* samples using a RNAprep Pure Plant Kit (Tiangen Biotech, Beijing, China). After extracting total RNA from individual plant samples, the RNA samples were assessed for purity, concentration, and integrity using a NanoDrop 2000 spectrophotometer (Thermo Scientific, Waltham, MA, USA) and an Agilent 2100 Bioanalyzer (Agilent Technologies, Palo Alto, CA, USA). Library preparation and sequencing were conducted at the BioMarker (Qingdao, China). The procedures followed included mRNA enrichment, fragmentation, first- and second-strand cDNA synthesis, end repair, A-tailing, adapter ligation, and product purification. Indexed libraries underwent paired-end sequencing (2 × 150 bp) using an Illumina Novaseq 6000 platform. Raw reads were trimmed with Trimmomatic v0.39 to remove adapters and low-quality bases (Phred < 20) and retain reads longer than 75 bp [22,23]. De novo assembly was then performed on the clean reads using Trinity v2.15.1 [24]. The assembled contigs were compared against the GenBank database using BLASTx and BLASTn with an E-value threshold of ≤1 × 10^−5^.

### 2.3. Molecular Detection, Rapid Amplification of cDNA Ends, and Full Viral Genome Amplification of Viruses

Total RNA was extracted from ten *G. bicolor* samples using a TaKaRa MiniBEST Plant RNA Extraction Kit (Takara, Dalian, China). For each *G. bicolor* sample, 100 mg of tissue was used. After total RNA was extracted from a single plant, cDNA synthesis was performed using an M5 Sprint qPCR RT Kit. (Mei5 Biotechnology, Beijing, China). PCR reactions (20 μL) contained two μL cDNA, 10 μL 2× M5 HiPer Taq HiFi PCR mix, one μL forward primer, one μL reverse primer, and six μL ddH_2_O. The 5′ and 3′ terminal sequences of the viral genome were amplified using a SMARTer^®^ RACE 5′/3′ Kit (Takara Bio, Dalian, China) according to the manufacturer’s instructions. The primers used to amplify these DNA fragments are listed in Supplementary Table S1. The PCR fragments were treated with pTOPO-TA (Aidlab, Beijing, China) and submitted for sequencing to Sangon Biotech (Beijing, China).

### 2.4. Detection of Virions by Transmission Electron Microscopy

The leaves and stem tissues of *G. bicolor* samples were homogenized in 0.01 M PBS buffer (pH 7.4) at a 1:10 *w/v* ratio. The homogenate was centrifuged at 9000× *g* for 3 min. Ten microliters of supernatant were loaded onto carbon-coated copper grids (400 mesh). The grids were incubated at room temperature for 5 min. Excess liquid was removed with filter paper. A 2% phosphotungstic acid solution (pH 7.0) was applied for negative staining. The sample was stained for 15 min, and the solution was blotted. The grids were dried at room temperature. The samples were observed using a Hitachi H-7650 transmission electron microscope (Hitachi High Technologies, Tokyo, Japan) at an accelerating voltage of 80 kV.

### 2.5. Viral Sequence Analysis

The viral open reading frames (ORFs) were predicted using Vector NTI Advance^®^ 11.5. Conserved domains of the virus were identified using the NCBI Conserved Domain Database (CDD). Multiple sequence alignments were conducted in MEGA12 using the ClustalW algorithm [25]. Phylogenetic analysis was performed using the maximum likelihood method in MEGA12, with the best-fit substitution model determined by ModelFinder. Tree topology confidence was evaluated with 1000 bootstrap replicates. The phylogenetic tree was visualized and annotated using ChiPlot (https://www.chiplot.online/). Pairwise nucleotide and amino acid identity percentages were calculated using SDT v1.2.

### 2.6. Quantitative RT-PCR of Viral RNA

Quantitative RT-PCR was performed using a Q2000B real-time PCR system (LongGene, Hangzhou, China). Each 20 μL reaction contained 2× SGExcel FastSYBR Mixture (Sangon Biotech, Shanghai, China). Primers were designed for the viral coat protein (CP) and RNA-dependent RNA polymerase (RdRp) genes. The actin gene of *G. bicolor* samples was employed as an internal reference. Relative viral RNA expression levels were calculated using the 2^−ΔΔCt^ method. Each assay included three biological replicates and three technical replicates to ensure reproducibility.

### 2.7. Transmission Studies

Under greenhouse conditions, *Cuscuta chinensis* seeds were planted in 21 cm pots containing a soil–manure mix to promote vine growth. Mature dodder vines were manually wound around virus-infected *G. bicolor* plants confirmed positive by RT-PCR. After 15 days, infected dodder segments were transferred to healthy *Nicotiana occidentalis*, *Oenothera biennis*, and *Chenopodium album*. Virus-free dodder segments, confirmed by RT-PCR, were similarly mechanically inoculated to new sets of the same species as negative controls. All inoculated plants were monitored for 30 days for symptom development.

## 3. Results

### 3.1. Identification of Two Novel Viruses in G. Bicolor Through High-Throughput Sequencing Technology

Leaf samples with chlorosis and mottling symptoms were collected from 10 *G. bicolor* plants, mixed, and sequenced using an Illumina NovaSeq 6000 platform. The raw sequencing data underwent adapter trimming and quality filtering, yielding 48,639,523 high-quality reads. We assembled the high-quality reads using MEGAHIT and obtained 1845 contigs. Analysis of BLASTx and BLASTn revealed that 20 contigs were similar to vanilla distortion mosaic virus (VDMV, *Potyvirus vanillae*) isolate from *Coriandrum sativum* (GenBank accession: NC_025250). These 20 contigs showed 74.4–100% sequence identity with VDMV. The longest viral-associated contig (10,054 bp) spanned 85% (8554/9553 bp) of the VDMV genome, sharing 81.18% nucleotide identity. In addition, 14 contigs shared 35.0% to 82.5% nucleotide identity with the glehnia littoralis marafivirus (GLMV) isolate from *Glehnia littoralis* (GenBank accession: BK013331). The longest contig (6761 bp) mapped 24% of its nucleotides with the GLMV genome and exhibited 74.23% sequence identity. In this study, we provisionally designated the putative novel virus as gynura bicolor maculavirus (GBMV).

### 3.2. Sequence Determination and Structural Characterization of the Complete RNA Genomes of Two Viruses

In this study, ten RNA samples using RT-PCR identified VDMV and GBMV in all samples. RNA extracted from a mix of ten G. bicolor samples was subjected to RT-PCR and rapid terminal amplification. Sequencing and assembly of the amplicons yielded complete genomes of the two viruses. TA cloning, Sanger sequencing, and sequence assembly yielded two full-length genomes measuring 9981 nt and 6761 nt, respectively. BLASTN analysis showed that the 9981-nt genome shared 81.13% identity with the VDMV isolate VDMV-Cor. The 6761-nt genome shared 74.24% identity with GLMV. These results indicated that the two viruses represented a new VDMV strain and a novel member of the family *Tymoviridae*.

**Figure 1:**
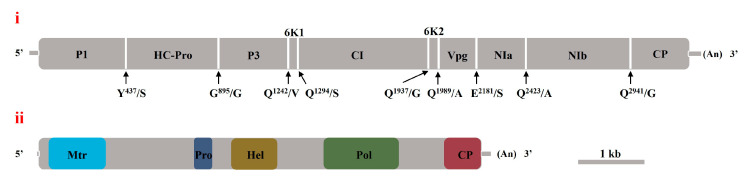
Schematic diagram of viral genome structural characteristics. (**i**) The genomic structural characteristics of VDMV isolate 23HN_GyBi1 from G. bicolor. Arrows indicated predicted dipeptide cleavage sites on the viral polyprotein. (**ii**) The genomic structural characteristics of GBMV.

This study analyzed the genomic structures of VDMV and GBaV using Vector NTI to predict ORFs and CDD to identify conserved protein domains. The VDMV isolate from G. bicolor contained a single ORF from nt 149 to 9805, encoding a 3218 aa polyprotein. This ORF was flanked by 5′ and 3′ untranslated regions. CDD analysis revealed 10 conserved domains (E-value < 0.01) within the polyprotein. These domains included potyvirus P1 protease (nt 686–1456), helper component proteinase (nt 1526–2833), and protein P3 of the potyviral polyprotein (nt 2870–4183). Other domains included the DEAD-like helicases superfamily (nt 4238–4699), the helicase conserved C-terminal domain (nt 4763–5110), and the potyviridae polyprotein (nt 5183–6025). Peptidase family C4 (nt 6686–7381), catalytic core domains of RdRp (nt 7769–8023; 7901–8608), and the potyvirus coat protein (nt 9101–9799) were also identified. The viral proteases cleaved this polyprotein into 10 distinct products. This study predicted dipeptide cleavage sites within the viral polyprotein through sequence comparison with the VDMV isolate VDMV-Cor (Figure 1i).

The genomic architecture of GBMV resembled that of the members of the genus *Marafivirus* (Figure 1ii). The 5′ UTR of the GBMV genome comprised 39 nt, followed by an ORF from nt 40 to 6609. This ORF encoded a 240.8 kDa polyprotein containing six virus-related conserved domains. These domains included viral methyltransferase (nt 151–999), salyut domain (nt 1480–1611), and tymovirus endopeptidase (nt 2272–2541). They also included superfamily 1 RNA helicase (nt 2839–3528), RdRp catalytic core (nt 4255–5373), and tymovirus coat protein (nt 6061–6603). Proteases processed the polyprotein into five protein products. The 3′ UTR spanned 158 nt. However, the characteristic marafibox sequence [CA(G/A)GGUGAAUUGCUUC], functioning as a subgenomic RNA promoter, was absent from the genome sequence of GBMV.

### 3.3. Sequence Identity Analysis and Phylogenetic Analysis

**Figure 2:**
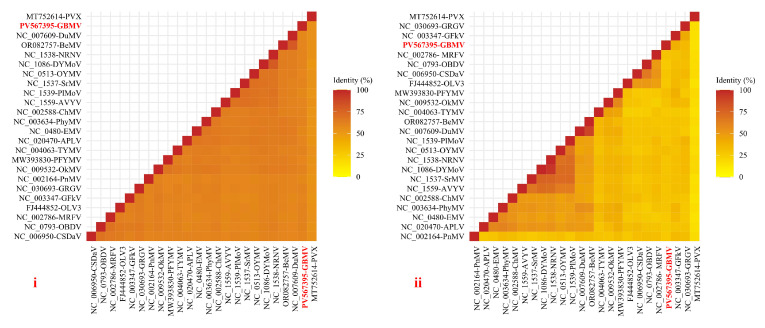
Sequence identity analysis based on whole-genome nucleotide sequence (**i**) and CP gene amino acid sequences (**ii**) of GBMV and representative isolates of all tymovirids.

Sequence identity analysis showed that VDMV isolate 23HN_GyBi1 shared 80.98% nucleotide identity and 86.79% amino acid identity with the polyproteins of the known VDMV. These values exceeded the International Committee on Taxonomy of Viruses (ICTV) threshold for new species of the genus *Potyvirus* (76% for nucleotides, 82% for amino acids). Therefore, they belonged to the same species. In contrast, GBMV showed 42.0–58.2% the whole genome nucleotide identity and 13–39% the amino acid identity of CP with other tymovirids (Figure 2). The obtained values are substantially lower than the ICTV demarcation criteria for the family *Tymoviridae*, defined as less than 80% overall genome sequence identity or less than 90% capsid protein sequence identity. Therefore, GBMV is a novel species within the family *Tymoviridae*.

**Figure 3:**
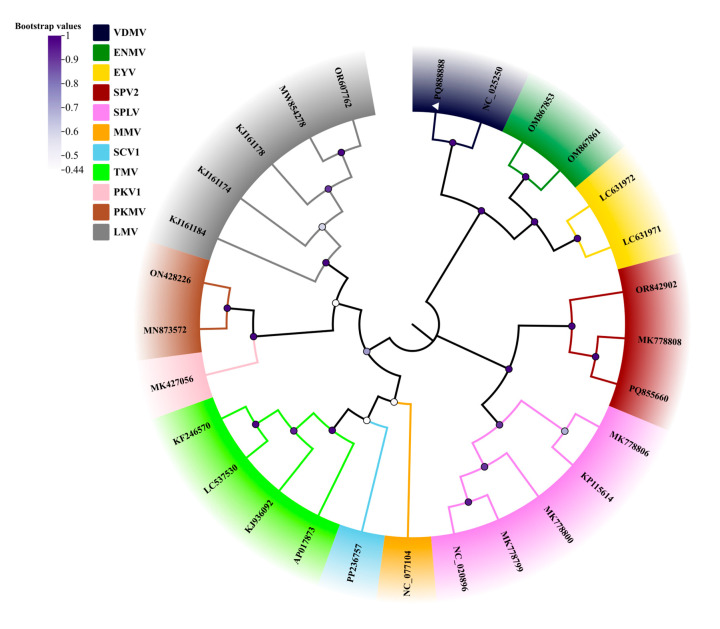
The phylogenetic tree was inferred using the ML method implemented in MEGA v. 12 software, based on whole-genome nucleotide sequences of VDMV and selected potyviruses. The LG + G + I model was chosen as the optimal nucleotide substitution model, and nodal support was assessed with 1000 bootstrap replicates.

**Figure 4:**
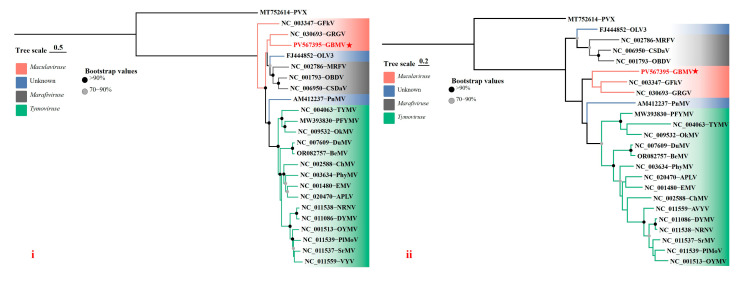
Phylogenetic trees were inferred using the maximum likelihood method implemented in MEGA v.12 software, based on the RdRp (**i**) and CP (**ii**) amino acid sequences of GBMV and representative isolates of all Tymovirids. Branch support was assessed with 1000 bootstrap replicates. The best-fit substitution models were determined to be General Reverse Transcriptase + G+ I+ F for the RdRp genes and the Le and Gascuel +G models for the CP genes.

To clarify the taxonomic status of the two viruses identified in *G. bicolor*, phylogenetic trees were reconstructed using the ML method implemented in MEGA12. Polyprotein amino acid sequences of VDMV, along with those of representative species within the genus, were used for analysis. In the resulting tree, the VDMV isolate 23HN_GyBi1 from *G. bicolor* clustered tightly with the VDMV isolate VDMV-Cor from *C. sativum*, indicating a close genetic relationship (Figure 3). Phylogenetic analyses based on CP and RdRp amino acid sequences, including GBMV and representative species of the family, consistently grouped GBMV within the genus *Maculavirus* (Figure 4).

### 3.4. Transmission Electron Microscopy Observations and Quantification of Two Viruses in Roots, Stems, and Leaves

**Figure 5:**
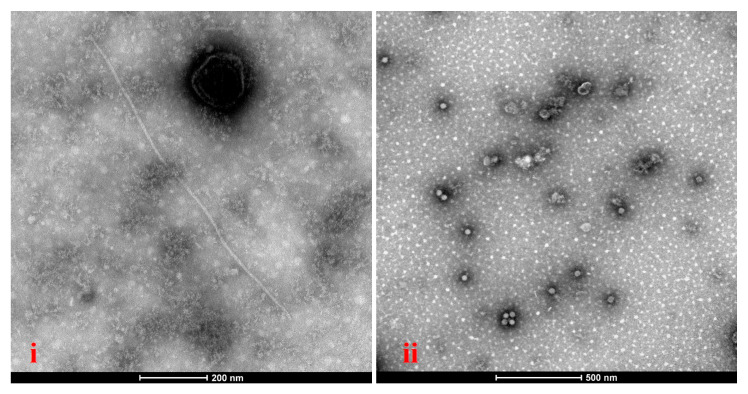
TEM observed the virions in the *G. bicolor* samples. (**i**) Virus particles observed in the leaf tissues of the *G. bicolor* samples. (**ii**) Virus particles observed in the stem tissues of the *G. bicolor* samples.

**Figure 6:**
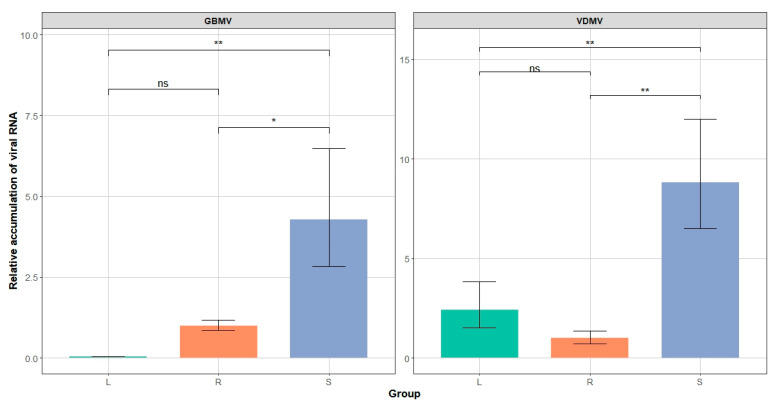
Accumulation of VDMV and GBMV RNA in different tissues of the *G. bicolor* samples. L, R, and S denoted the leaves, roots, and stems of the *G. bicolor* samples, respectively. ns = not significant; * *p* < 0.05; ** *p* < 0.01.

Transmission electron microscopy (TEM) of the stems and leaves from the G. bicolor samples infected with GBMV and VDMV revealed distinct virion types (Figure 5). The stems contained linear virions (650–960 nm) and isometric virions (~30 nm), whereas the leaves displayed only linear virions. Quantitative RT-PCR quantified VDMV and GBMV loads in the roots, stems, and leaves (Table S2). Two viruses reached their highest abundance in the stems (Figure 6). VDMV was more abundant in the leaves than in the roots, whereas GBaV occurred at extremely low levels in the leaves.

### 3.5. Doddertransmission

**Figure 7:**
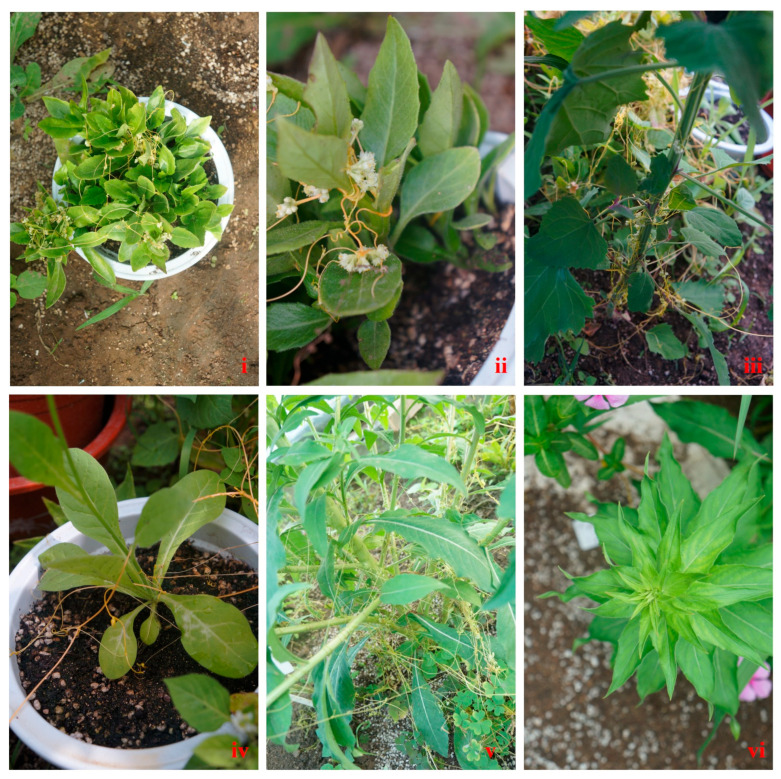
Dodder transmission (**i**,**ii**), *Cuscuta chinensis* established on infected *G. bicolor* (**iii**), infected *Cuscuta chinensis* wrapped on *C. album* (**iv**), infected *Cuscuta chinensis* wrapped on *N. occidentalis* (**v**), infected *Cuscuta chinensis* wrapped on *O. biennis* (**vi**). *O. biennis* exhibited symptoms of chlorosis and mosaic after being wrapped by infected *Cuscuta chinensis*.

After 15 days of wrapping Cuscuta around *G. bicolor* infected with GBMV and VDMV, both viruses were detected in *Cuscuta chinensis* by RT-PCR (Figure 7i). Then, the virus-carrying *Cuscuta chinensis* was wrapped around healthy plants of *N. occidentalis*, *O. biennis*, and *C. album* for 15 days. RT-PCR detected both viruses in all three plants. Notably, *O. biennis* infected with both viruses showed chlorosis and mottling symptoms (Figure 7vi).

## 4. Discussion

High-throughput sequencing reliably identifies unknown plant viruses and yields near-complete genome sequences [26]. Through high-throughput sequencing (HTS), we identify the presence of VDMV and a novel maculavirus, tentatively named GBMV, in chlorotic and leaf-curling samples of *G. bicolor*. We have obtained the complete genome sequences of VDMV and GBMV through RT-PCR. GBMV’s genome differs from known maculaviruses but resembles oat blue dwarf virus (OBDV, *Marafivirus avenae*), containing a single long ORF, suggesting possible recombination events [27,28]. VDMV occurs only in China and India, infecting multiple hosts, including *Zinnia bicolor*, *Vanilla planifolia*, *Coriandrum sativum*, *Cuminum cyminum*, *Daucus carota*, and *Stevia rebaudiana* [29]. The polyprotein of the VDMV isolate 23HN_GyBi1 shares 81% nucleotide and 87% amino acid identity with known isolates, indicating a marked divergence. Its longer, variable N-terminal region, common among potyviruses, may reflect host-specific adaptation. We classify 23HN_GyBi1 as a new VDMV strain.

Mixed infections involving the members of the genus potyvirus and heterologous viruses are widespread [12,13]. This study, using HTS and RT-PCR methods, reveals that ten *G. bicolor* samples are infected with a potyvirus and a novel maculavirus. However, electron microscopy reveals only filamentous particles in leaf tissues. In addition, qRT-PCR assays indicate that GBMV accumulates at a minimal viral RNA level in leaf tissues. These findings suggest limited plasmodesmatal connectivity or stronger RNA silencing in photosynthetic tissues [30]. This study detects no *G. bicolor* sample individual infected with either VDMV or GBMV; thus, viral titers between single and mixed infections are not compared. Nevertheless, the biological and epidemiological consequences of mixed-virus infections are inherently unpredictable [31,32]. For example, co-infection of potato virus A (PVA, *Potyvirus atuberosi*) and potato leafroll virus (PLRV) allows PLRV, a phloem-restricted virus, to invade mesophyll tissue. Similarly, co-infection of sweet potato chlorotic stunt virus (SPCSV, *Crinivirus ipomeae*) and sweet potato feathery mottle virus (SPFMV, *Potyvirus batataplumei*) enhances SPFMV replication, causing sweet potato virus disease (SPVD), which is characterized by chlorotic leaves, distorted growth, and stunted plants [31,32]. Thus, further research is warranted to investigate the synergistic mechanisms between GBMV and VDMV.

For plant viruses, the key to successfully invading a host lies in their ability to enter the vascular phloem and achieve systemic spread [33]. In contrast, dodder haustoria can directly connect to the host’s vascular system, creating a bidirectional pathway for the transport of viruses and nutrients [34]. Therefore, we have employed the method of the dodder entanglement to transmit GBMV and VDMV to healthy plants of *N. occidentalis*, *O. biennis*, and *C. album*. After 15 days of coiling with GBMV- and VDMV-infected dodder, both viruses have been detected in these hosts. Notably, healthy *O. biennis* exhibits leaf chlorosis 30 days after infection with GBMV and VDMV, indicating a substantial transmission risk for these viruses. Consequently, it is essential to focus on predicting and providing early warnings for GBMV and VDMV during the production of *G. bicolor*, and to strengthen field management measures against their viral diseases.

## Data Availability

The genome sequences of Vanilla distortion mosaic virus (VDMV) and GBaV have been deposited in the NCBI GenBank database with the accession numbers PV567394 and PV567395, respectively.

## Supplementary Materials

The following supporting information can be downloaded at: https://www.mdpi.com/article/10.3390/v17101290/s1, Table S1: Primers Used in This Study; Table S2: Quantitative PCR Ct values for VDMV, GBMV, and the internal control gene (Actin) across various plant organs.
