# Supplementary material for: Coinfection of Gynura bicolor with a New Strain of Vanilla Distortion Mosaic Virus and a Novel Maculavirus in China
Source: Viruses. 2025 Sep 24;17(10):1290. doi: 10.3390/v17101290 (PMC12567806; doi:10.3390/v17101290)
Supplement: Supplementary file 1 [file viruses-17-01290-s001.zip › viruses-3838306-supplementary.pdf]

Supplementary Table S1 Primers Used in This Study

	Sequence (5′ to 3′)	Amplicon size (bp)	Purpose
ZBN1-1	AGAATGAGGAACTTGAACGACAT	299/**422**	Molecular Detection and **3′-RACE**
ZBN1-2	TAGTTCTGTTCGGTTGAGTCAT		Molecular Detection
ZBN1-3	CTATCTTCATGGGTCACTTGCT	666	5′-RACE
ZBN1-4	ACTTCTTGCTTCTCCTCCTCCT	477	5′-RACE
ZBN1-5	AGAATACGAGGTGGATGATAGC	177/**681**	Quantitative RT-PCR and **3′-RACE**
ZBN1-6	TGTCTGAAGGTTGGTTGTGCAT		Quantitative RT-PCR
ZBN1-7	GTGTGTTTTGCAAACGGGATAT	3129	Full-genome sequence amplification
ZBN1-8	GCGTTACGTTCGATGATTGCAT		Full-genome sequence amplification
ZBN1-9	TGATATCTCCAAGTGTCGTCAT	3375	Full-genome sequence amplification
ZBN1-10	CTGGGTCGTATCCATACATGT		Full-genome sequence amplification
ZBN1-11	AGATGACAAAACAATCGAGCACT	3789	Full-genome sequence amplification
ZBN1-12	GTCTCTAGGCTTCCATGGACAAG		Full-genome sequence amplification
ZBN2-1	CCACCTGTTCTCGAGCACCCT	339	Molecular Detection and Quantitative RT-PCR
ZBN2-2	GATCAGATCTGATCGATGAGGT		Molecular Detection
ZBN2-3	GAGAAGTCGGATTCAGGAGGAAT	575	5′-RACE
ZBN2-4	CATTAGTGAGAAGGTTGAGTTCT	280	5′-RACE
ZBN2-5	ACACCTATGGGTCCACTAGGAT	409	3′-RACE
ZBN2-6	GTGACAATCCCATCCTCTACAT	292	3′-RACE
ZBN2-7	ATCAGAGGTGGAGGAGGATGAT	189	Quantitative RT-PCR
ZBN2-8	AGTCATAGCGTACTTTTCTTAC	3618	Full-genome sequence amplification
ZBN2-9	TTGTAGCTCGACGGTCTGACCT		Full-genome sequence amplification
ZBN2-10	CACCATAGCCAGTTCTCAAGG	3357	Full-genome sequence amplification
ZBN2-11	ATCCGGGAAACTAAAAGCTTAAG		Full-genome sequence amplification

Supplementary Table S2 Quantitative PCR Ct values for VDMV, GBMV, and the internal control gene (Actin) across various plant organs.

Plant Organ	Target Gene	Ct Value 1	Ct Value 2	Ct Value 3	Mean ± SD
Root	Actin	18.34	18.30	18.38	18.34 ± 0.04
Stem	Actin	20.59	20.54	20.64	20.59 ± 0.05
Leaf	Actin	18.47	18.43	18.51	18.47 ± 0.04
Root	VDMV	14.33	14.01	14.03	14.12 ± 0.17
Stem	VDMV	13.44	13.16	13.10	13.23 ± 0.18
Leaf	VDMV	13.14	13.14	12.68	12.99 ± 0.27
Root	GBMV	19.99	19.96	20.14	20.03 ± 0.09
Stem	GBMV	20.04	20.04	20.45	20.18 ± 0.24
Leaf	GBMV	24.71	24.70	24.75	24.72 ± 0.03
